# Isolation and analysis of sugar nucleotides using solid phase extraction and fluorophore assisted carbohydrate electrophoresis

**DOI:** 10.1016/j.mex.2016.03.010

**Published:** 2016-03-14

**Authors:** Jarrod Barnes, Liping Tian, Jacqueline Loftis, James Hiznay, Suzy Comhair, Mark Lauer, Raed Dweik

**Affiliations:** a Departments of Pathobiology, Cleveland Clinic, 9500 Euclid Ave, Cleveland, OH 44195, USA; b Pulmonary Critical Care Medicine, Respiratory Institute, Cleveland Clinic, 9500 Euclid Ave, Cleveland, OH 44195, USA; c Molecular Medicine, Cleveland Clinic, 9500 Euclid Ave, Cleveland, OH 44195, USA

**Keywords:** HPLC, high performance liquid chromatography, SPE, solid phase extraction, FACE, fluorophore assisted carbohydrate electrophoresis, UDP, uridine diphosphate, GDP, guanosine diphosphate, CMP, cytosine monophosphate, TEAA, triethylamine acetate, APS, ammonium persulfate, Gal, galactose, GalNAc, N-acetylgalactosamine, GlcNAc, N-acetylglucosamine, Man, Mannose, NeuAc, sialic acid, GlcUA, glucuronic acid, AMAC, 2-aminoacridone, TEMED, *N*′,*N*′,*N*′*N*′-tetramethylenediamine, Sugar nucleotide analysis by SPE and FACE, Sugar nucleotides, Face, HPLC, Carbohydrate, Electrophoresis

## Abstract

The building blocks of simple and complex oligosaccharides, termed sugar nucleotides, are often overlooked for their role in metabolic diseases and may hold the key to the underlying disease pathogenesis. Multiple reasons may account for the lack of analysis and quantitation of these sugar nucleotides, including the difficulty in isolation and purification as well as the required expensive instrumentation such as a high performance liquid chromatography (HPLC), mass spectrometer, or capillary electrophoresis. We have established a simple yet effective way to purify and quantitate sugar nucleotides using solid phase extraction (SPE) chromatography combined with fluorophore assisted carbohydrate electrophoresis (FACE). The simplicity of use, combined with the ability to run multiple samples at one time, give this technique a distinct advantage over the established methods for isolation and analysis of sugar nucleotides from cell culture models.

• Sugar nucleotides can be easily purified with solid phase extraction chromatography.
• FACE can be used to analyze multiple nucleotide sugar extracts with a single run.
• The proposed method is simple, affordable, and uses common everyday research labware.

## Graphical abstract

**Figure:**
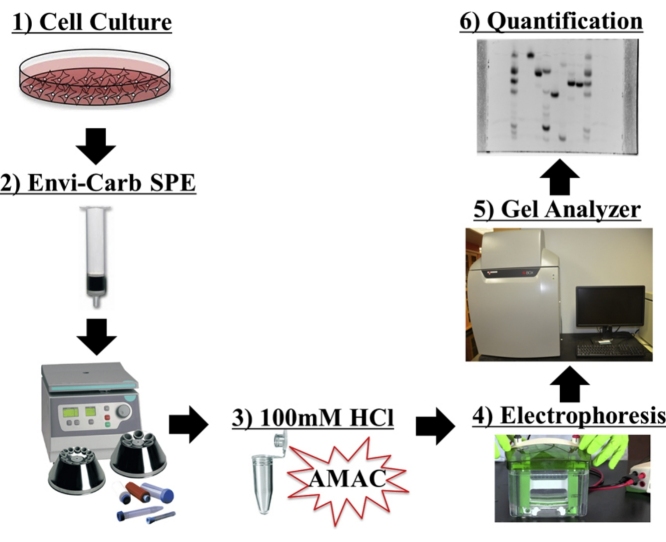

## Method details

The biosynthesis of polysaccharides and the N- and O-linked glycosylation of proteins depend the charged substrates of glycosyltransferases called sugar nucleotides. These substrates are sugars activated by the addition of a nucleoside mono- or diphosphate (UDP, GDP, or CMP), which forms the sugar nucleotide. A large proportion of the sugar nucleotides are synthesized in the cytosol, while their respective enzymes are located within the lumen of the Golgi apparatus or the endoplasmic reticulum. Thus, sugar nucleotides are translocated from the cytosol to the lumen of the Golgi apparatus and endoplasmic reticulum by multiple spanning domain proteins known as sugar nucleotide transporters. It is in these compartments that glycoconjugates, including polysaccharides, glycoproteins, and glycolipids are synthesized and glycosylated by glycosyltransferases. Glycosylation and their respective glycosyltransferases have become the predominant focus of the literature when glycan alterations are investigated in growth, development, and disease processes such as cancer and pulmonary arterial hypertension [1], [2], [3], [4], [5], [6] with little focus on the role of nucleotide sugars.

Sugar nucleotides were first discovered by Leloir and colleagues [7], [8], [9]. It is now known that the vast majority of sugar nucleotides is derivatized from UDP-glucose (glc); a reaction that take places predominantly in the cytosol and gives rise to several sugar nucleotides including UDP-galactose (Gal), UDP-N-Acetylglucosamine (GlcNAc), UDP-N-Acetylgalactosaime (GalNAc), GDP-Mannose (Man), and CMP-Sialic Acid (NeuAc). Multiple reports have demonstrated methods that generate or synthesize sugar nucleotides [14], [15], [16], [17], [18], [19], [20] as well as high-throughput assays for sugar nucleotide formation and glycosyl transfer [21], [22]. Others have shown great promise with the use of sugar nucleotide derivatives and sugar analogs for the inhibition of glycosyltransferases [22], [23], which may be useful in disease such as cancer. Since the late 1970s, several HPLC methods have been developed to purify and analyze sugar nucleotides [10], [11], [12], [13]. In addition, Mass Spectrometry [11], [24], [25], capillary electrophoresis [26], [27], and NMR methods [11], [28], [29] have been put forth to study sugar nucleotide levels. However, these instruments are expensive and require extensive expertise to operate.

**Fig. 1:**
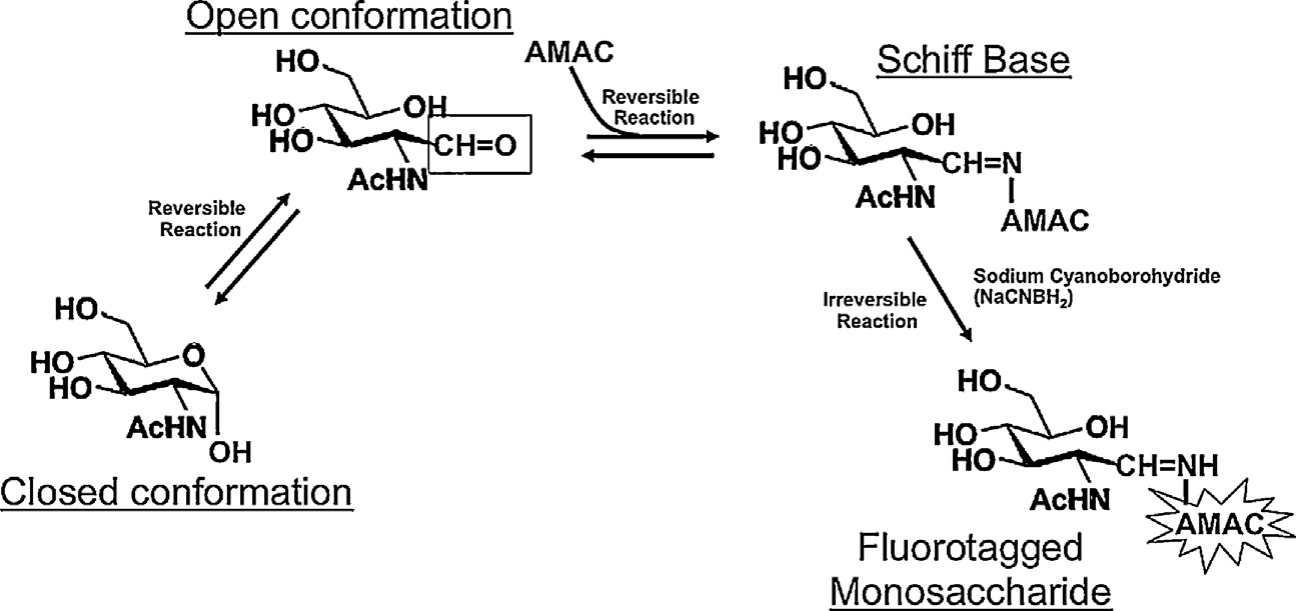
AMAC conjugation forms a stably fluorescent-labeled monosaccharide. AMAC performs a nucleophilic attack on the carbonyl (C-1) carbon of a reducing sugar and forms a Schiff's base. The resulting imine group is reduced using sodium cyanoborohydride to yield a fluorotagged monosaccharide. Abbrv. AMAC = 2-aminoacridone. Adapted from [34].

Fluorophore Assisted Carbohydrate Electrophoresis (FACE) was created as a simple alternative to Mass Spectrometry (MS), NMR, and HPLC for determining carbohydrates and oligosaccharides [30], [31], [32], [33], [34]. Two different fluorophores, 8-aminonaphthalene- 1,3,6-trisulphonic acid (ANTS) and 2-aminoacridone (AMAC), have routinely been used to fluorescently label carbohydrates (Fig. 1) for visualization [30], [31]. There are a few studies that have used FACE to analyze sugar nucleotides in combination with anion exchange chromatography [35], [36]. However, the anion exchange columns also bind monosaccharides modified with phosphates and other charged entities as well as oligosaccharides. Therefore, multiple steps are required to purify the sugar nucleotides from these other glycans.

Previously published reports have shown that the Solid Phase Extraction (SPE) ENVI-carbon columns bind tightly to sugar nucleotides and not other monosaccharides or glycoconjugates [12], [13]. Bound sugar nucleotides can be eluted using an ion-pairing reagent such as TEAA [12], [37]. Interestingly, Markku Tammi and colleagues have shown a substantial yield of the sugar nucleotides using SPE combined with HPLC, which was greater than 89 percent [13].

In this report, we show that the combination of SPE chromatography, mild acid hydrolysis, and FACE can be used to simply and effectively purify, analyze, and quantitate sugar nucleotides from cell extracts. The advantages of this method compared to HPLC, NMR, and MS are cost affordability, the use of common labware, and the analysis of multiple samples in a single run. We believe that this method is a simple, yet affordable approach to analyzing sugar nucleotides from cultured cells.

## Methods

### Reagents

• PBS (cat # 70011-044; ThermoFisher, Grand Island, NY, USA)
• 75.0% ice cold ethanol (cat # 459836; Sigma, St. Louis, MO, USA)
• ENVI-Carb SPE column (cat # 57109-U; Sigma, St. Louis, MO, USA)
• Acetonitrile (cat # 34998; Sigma, St. Louis, MO, USA)
• Trifluoroacetic acid (cat # 302031; Sigma, St. Louis, MO, USA)
• Ammonium bicarbonate (cat # A6141; Sigma, St. Louis, MO, USA)
• Triethylamine acetate (TEAA) pH 7.0 (cat # 90357; Sigma, St. Louis, MO, USA)

#### Protocol

##### Sugar nucleotide extraction from cultured cells

1 Wash cells with ice cold PBS and collect by scraping with a rubber policeman into a 2 mL eppendorf tube.
2 Centrifuge cells at 10,000 × *g* (4 °C) for 10 min and fix with 75.0% cold ethanol.
3 Sonicate (10 pulses, 1 s each with 10.0% amplitude) on ice in the 75% ethanol using a 130-Watt Pulse Ultrasonic Processor (cat # 9655A09; Thomas Scientific, Swedesboro, NJ, U.S.A.).
4 Remove debris and ethanol insoluble cell material by centrifugation (16,000 *×* *g* for 10 min at 4 °C).
5 Discard precipitates and dry ethanol soluble supernatants using a Speed-Vacuum Dryer.
6 Store the dry supernatant in the −20 °C freezer if it is ready before the column is conditioned.

Note: After collecting cells from step 1, reserve 80 μL of cells for protein assay or DNA quantitation (Quant-iT™ PicoGreen^®^ dsDNA Assay Kit; cat # P11496, ThermoFisher, Grand Island, NY, USA) following manufacturer’s protocol. Spin and discard supernatant and keep cell precipitate and store in the −80 °C freezer.

#### Purification of sugar nucleotides by Solid-Phase Extraction (SPE) Chromatography

1 Place a 1 mL/100 mg ENVI-Carb SPE column in a 15 mL conical tube.
2 Equilibrate the column by adding 1 mL of 80% acetonitrile in 0.1% trifluoroacetic acid and spin at 60 × *g* for 45 s (room temperature). Repeat twice.
3 Add 1 mL of ultrapure water to the column and spin as described in step 1. Repeat once.
4 Reconstitute the dried cell samples (generated from Sugar nucleotide extraction from cultured cells, step 6) in 2 mL of 10 mM ammonium bicarbonate.
5 Add 1 mL of the dissolved sample to the column and spin as in step 1. Repeat with the other half of the sample.
6 Collect flow-through and enrich by re-applying the sample to the ENVI-Carb column in 1 mL fractions (same as step 5).
7 Wash the column with 2 mL of ultrapure water, 2 mL of 25% acetonitrile, 1 mL of ultrapure water, and 2 mL of 10 mM TEAA buffer (pH 7) and spin after each wash as described in step 2.
8 Collect SPE Envi-Carb column(s) and place into a new 15 mL conical tube.
9 Elute the bound sugar nucleotides with 2 mL of 25% acetonitrile in 50 mM TEAA buffer (pH 7). Spin and pool as described in step 2.
10 Transfer the purified sugar nucleotides to a 2 mL eppendorf tube.
11 Evaporate eluted fractions to dryness using a SpeedVac concentrator to remove the TEAA.

Note: Recovery of sugar nucleotides from SPE Envi-Carb columns using TEAA buffer as the elution solvent has been reported and used in combination with HPLC [13].

#### Mild Acid Treatment of UDP-Sugars to release monosaccharide from nucleotide

**Fig. 2:**
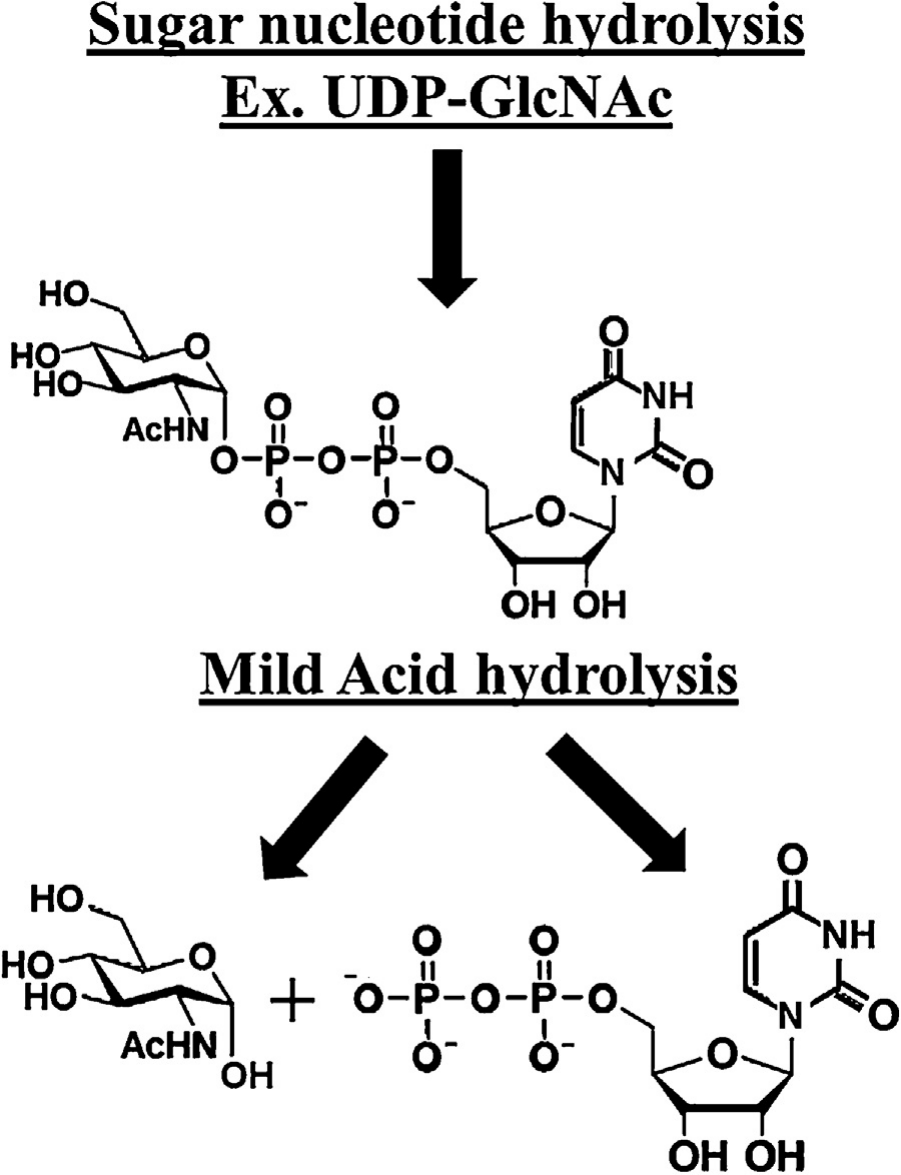
Schematic representation of the mild acid (100 mM HCl) hydrolysis of sugar nucleotides. Mild acid addition leads to hydrolysis of the carbonyl carbon (C-1) and phosphate bond, which results in a free monosaccharide and a nucleoside diphosphate [38]. This hydrolysis results in a reducing sugar that is now ready for 2-aminoacridone (AMAC) conjugation (Fig. 1) and FACE analysis.

1 Resuspend the dried samples in 50 μL of a 50 mM HCl solution and boil at 100 °C for 20 min to hydrolyze the nucleotide from the sugar monosaccharide (Fig. 2) [38].
2 Acid hydrolyzed samples are then dried using a Speed-Vacuum concentrator.

**Protocol 2.2**

#### Reagents

• AMAC [(2-aminoacridone); cat # A-6289; Molecular Probes, ThermoFisher, Grand Island, NY, USA)].
• Sodium cyanoborohydride (cat # 156159 Sigma, St. Louis, MO, USA).

#### Prepare acid hydrolyzed monosaccharides for aminoacridine (AMAC) conjugation

1 Prepare a 12.5 mM solution of AMAC in 15% (v/v) acetic acid.
2 Prepare a 1.25 M solution of sodium cyanoborohydride in dimethyl sulfoxide.
3 Prepare the AMAC by adding 15% glacial acetic acid to 12.5 mM AMAC.
4 Mix a 1:1 solution of AMAC/acetic acid with 1.25 M sodium cyanoborohydride to make ‘activated’ AMAC.
5 Reconstitute the acid hydrolyzed samples in 10 μL of the activated AMAC solution.
6 Incubate overnight at 37 °C using an orbital shaker (300 rpm) and covered in aluminum foil.
7 Prepare for FACE analysis or store samples in the −20 °C freezer.

Note: Both stock solutions of AMAC (12.5 mM) and sodium cyanoborohydride (1.25 M) and unused activated AMAC can be stored in the −80 °C freezer and re-used for up to two months.

#### Reagents

The purified monosaccharides: N-Acetylgalactosamine (GalNAc), Mannose (Man), Sialic Acid (NeuAc), N-Acetylglucosamine (GlcNAc), Glucuronic Acid (GlcUA), Galactose (Gal), Glucose (Glc) were all purchased from Sigma Aldrich, St. Louis, MO, USA.

#### Preparation of a monosaccharide standard

**Fig. 3:**
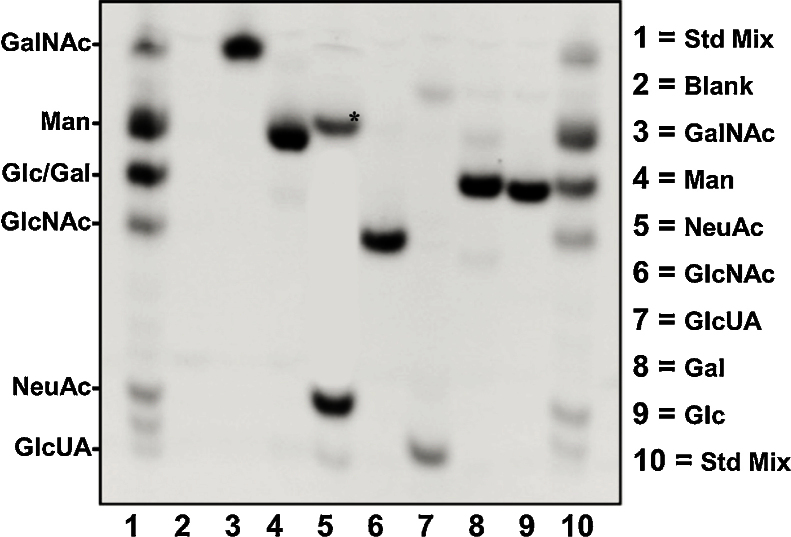
Generating a monosaccharide standard for sugar nucleotide analysis. 1 = Std Mix, Monosaccharide Standard Mixture; 2 = Blank; 3 = GalNAc, N-Acetylgalactosamine; 4 = Man, Mannose; 5 = NeuAc, N-Acetylneuraminic acid; 6 = GlcNAc, N-Acetylglucosamine; 7 = GlcUA, Glucuronic acid; 8 = Gal, Galactose; 9 = Glc, Glucose; 10 = Std Mix, Monosaccharide Standard Mixture. Asterisk denotes a small amount of mannose impurity in the sialic acid mixture.

1 Monosaccharides (listed above) were prepared and diluted (see below) in ammonium acetate buffer and dried as in Reagents.
2 The monosaccharides were mixed in an activated AMAC solution (10 μL total volume) and incubated overnight similar to step 6, Prepare acid hydrolyzed monosaccharides for aminoacridine (AMAC) conjugation.
3 Two microliters of each monosaccaride was then combined to make a monosaccharide standard mix.
4 For gel electrophoresis, 2 μL of the monosaccharide standard mix was loaded in the FACE gel (for FACE gel preparation see Casting gel for FACE & Resolving AMAC-conjugated monosaccharides using gel electrophoresis).
5 The mixed standards were run using FACE in parallel with the individual AMAC conjugated monosaccharides (2 μL each) to determine their respective electrophoretic mobility (Fig. 3).

**Monosaccharide Standard dilutions (final concentration):**

• GalNAc and GlcNAc = 1 μg/mL
• Man = 9.1 μg/mL
• Glc, Gal, and NeuAC = 6 μg/mL
• GlcUA = 3 μg/mL

Note: A known purified amount of UDP-GlcNAc was processed using this protocol and the percent yield was determined as ∼98.0% recovery (data not shown).

### Protocol

#### Equipment and reagents

• **40% 37.5:1 acrylamide solution** (cat # 161-0148; Bio-Rad, Hercules, CA, USA).
• **400** **mM Tris-Acetate Gel Buffer** (Sigma, Tris Base cat # T-6791) pH to 7.0 adjusted with glacial acetic acid.
• **10x TBE** (0.5 M Tris, 0.5 M Boric Acid, 10 mM EDTA, pH 8.3). Store 4 °C.
• 10% ammonium persulfate (APS).
• 100% *N*,*N*,*N*′*N*′-tetramethylenediamine (TEMED).

Note: We use the BioRad Mini-PROTEAN^®^ Tetra Module (cat # 1658004) and glass plates (cat # 1653310) with 0.75 mm spacers and combs (10- and 15-well). It is important to use a clear-white short plate (cat # GBW-101-73-1; Moliterno, Inc. Pepperell, MA, USA) because it improves imaging quality compared to other plates. Any comparable horizontal gel system will work.

Coil system (made in-house) with a Recirculating Chiller VWR Model 1162 (cat # 1699; Scientific Support, Inc., Hayward, CA, USA).

#### Casting gel for FACE

To prepare gel solution for casting for two (10 mL) resolving gels, combine 5 mL of a **40% 37.5:1 acrylamide solution** to 1.12 mL of the **400** **mM Tris-Acetate Gel Buffer pH 7.0.**

1 Add 0.250 mL of 100% glycerol and bring to 10 mL with 3.63 mL of H_2_O.
2 Add 50 μL of 10% APS and 10 μL of TEMED
3 Immediately pipette or decant resolving gel to a height of 0.5 cm below the bottom of the comb and overlay with 1 cm H_2_O.
4 Insert the 0.75 mm comb slowly to minimize the formation of air bubbles in the wells.
5 Remove and excess acrylamide off the plates that may have been squeezed out of the cast upon the addition of the comb.
6 Allow to polymerize for 10–15 min
7 Use immediately or place in 4 °C refrigerator or cold room and use within 1 day.

Note: Gels are cast using Tris-Acetate buffer pH 7. However, TBE buffer can also be used for casting gels with no substantial loss in band resolution or alteration in electrophoretic mobility (unpublished results).

#### Resolving AMAC-conjugated monosaccharides using gel electrophoresis

1 Pour cold 1X TBE buffer into the gel electrophoresis box and insert the cast gel into the electrode assembly apparatus and place in the box. Note: Be sure to position the electrode assembly with proper alignment (i.e., anode to anode and cathode to cathode).
2 Add cold 1X TBE to the electrode assembly apparatus and fill to the top.
3 Remove any acrylamide fragments left over from the casting with the comb by rinsing with a pipette tip. Note: acrylamide fragments in the wells may compromise the sample migration into the gel if not fully removed.
4 Add cold 1x TBE buffer to the gel box so that its level comes just below the bottom of the wells.
5 Place cooling coil, which is connected to the VWR-1152Chiller, into the gel box and begin circulating the anti-freeze solution (20% v/v methanol in H_2_O) through the coil to maintain the gel box and electrode assembly at 2–4 °C (run ∼30 min).
6 Load 1–2 μL of the AMAC conjugated sample in each well the gel
7 Load 2 μL of the monosaccharide ladder to a well (monosaccharide ladder prepared in Preparation of a monosaccharide standard).
8 Check the temperature in the gel box with a thermometer (2–4 °C).
9 Connect the gel electrophoresis assembly to a power supply and run at a constant current of 20 mA. Note: Continually check the temperature throughout the run. Adjust the circulation temperature as necessary to maintain the temperature at 2–4 °C.
10 Standards were run in parallel with the samples to help determine the electrophoretic mobility of the acid hydrolyzed/AMAC conjugated sugar nucleotides within the FACE gel (Fig. 3).
11 When the samples have fully separated, turn off the power supply and prepare for acquiring images for quantitation.

Note: You can check the migration of the AMAC-conjugated monosaccharides by pouring the buffer off from the upper chamber (pour in a beaker) and removing the gel cast from the electrode assembly. If it needs to run longer, pour the saved buffer back into the electrode assembly or use fresh (*do not mix the salts in the upper chamber with those in the lower chamber*).

#### Imaging the AMAC-conjugated monosaccharides

**Fig. 4:**
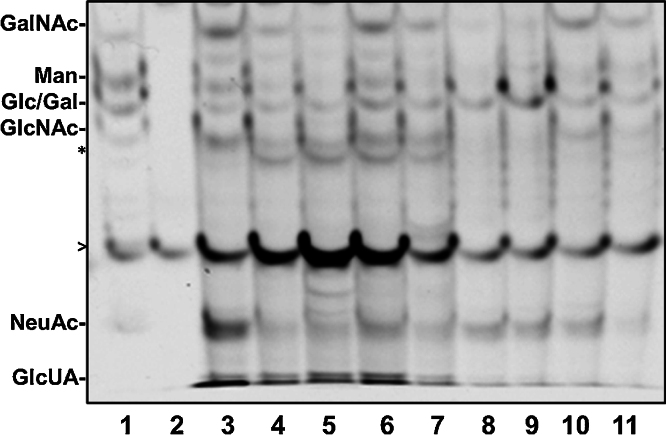
Sugar nucleotide analysis of multiple cell lines from cancer, pulmonary vascular disease, and metabolic disorders. 1 = Monosaccharide Standard; 2 = Blank; 3 = human colon adenocarcinoma grade II cells (HT29); 4 = human embryonic kidney (HEK) 293; 5 = human adenocarcinoma alveolar basal epithelial cells (A549); 6 = human cervical carcinoma epithelial cells (HeLa); 7 = type II diabetic human primary pulmonary arterial smooth muscle cells (PASMCs); 8 = IPAH human primary pulmonary arterial endothelial cells (PAECs); 9 = control human primary PAECs; 10 = control human primary PASMCs; and 11 = IPAH human primary PASMCs. Asterick denotes unidentified bands, while open arrow (>) denotes non-specific bands in the face gel. Abbrv: GalNAc, N-Acetylgalactosamine; Man, Mannose; NeuAc, N-Acetylneuraminic acid; Gal, Galactose; Glc, Glucose; GlcNAc, GlcUA, Glucuronic acid. IPAH = idiopathic pulmonary arterial hypertension. Primary isolation of PAECs and PASMCs were done as described [41].

In our hands, gel images were acquired (Fig. 4) using the G:BOX Chemi XR5 system and Gene Tools software v4.3.00. The densitometry of each AMAC conjugated sugar within the FACE gel can be determined using ImageJ software [39] and normalized to total cellular DNA (prepared in Sugar nucleotide extraction from cultured cells). However, there are many fluorescence imagers with CCD cameras available that are suitable for image acquisition.

## Conclusions

The isolation and purification of sugar nucleotides from cultured cells can be easily done using SPE and FACE. This method is direct, simple, and affordable for any researcher that has experience using everyday labware and common laboratory chemicals. In addition, the use of FACE is a safer alternative to radiolabeling as well as a cheaper and comparable method to SPE combined with HPLC [13]. This report also highlights a major advantage of the method whereby multiple samples from a single run can be analyzed on a gel (Fig. 4).

We have used FACE for quantitation of hyaluronan and other glycosaminoglycans involved in diseases such as idiopathic pulmonary hypertension (IPAH) and asthma [6], [40]. Furthermore, we have used the SPE and FACE method to determine the levels of UDP-GlcNAc in IPAH [5]. Future work will be aimed at determining the sugar nucleotide levels in multiple metabolic disorders such as IPAH, diabetes, and cancer (Fig. 4).

## Disclosures

None.

## Funding sources

This project was supported by the following grants from the National Institutes of Health: the Ruth L Kirschstein F32 postdoctoral fellowship (F32HL120629 to J.B.) and the Programs of Excellence in Glycosciences (1P01HL10714 to R.A.D.) both from NHLBI. In addition, primary human PAEC and PASMC isolation was supported by the translational Program Project Grant and the Program Project Grant P01HL103453 and P01HL081064, respectively (both to S.A.A.C and R.A.D). The content is solely the responsibility of the authors and does not necessarily represent the official views of the National Institutes of Health.
